# Clinical Impact of Physical Activity and Cough on Disease Progression in Fibrotic Interstitial Lung Disease

**DOI:** 10.3390/jcm12113787

**Published:** 2023-05-31

**Authors:** Tobias Veit, Michaela Barnikel, Nikolaus Kneidinger, Dieter Munker, Paola Arnold, Jürgen Barton, Alexander Crispin, Katrin Milger, Jürgen Behr, Claus Neurohr, Gabriela Leuschner

**Affiliations:** 1 Department of Internal Medicine V, Ludwig-Maximilian University Munich, 81377 Munich, Germany; tobias.veit@med.uni-muenchen.de (T.V.); michaela.barnikel@med.uni-muenchen.de (M.B.); nikolaus.kneidinger@med.uni-muenchen.de (N.K.); dieter.munker@med.uni-muenchen.de (D.M.); paola.arnold@med.uni-muenchen.de (P.A.); juergen.barton@med.uni-muenchen.de (J.B.); katrin.milger@med.uni-muenchen.de (K.M.); juergen.behr@med.uni-muenchen.de (J.B.); 2 Comprehensive Pneumology Center (CPC-M), German Center for Lung Research, 81377 Munich, Germany; 3 IBE—Institute for Medical Information Processing, Biometry and Epidemiology, Ludwig-Maximilian University, 81377 Munich, Germany; cri@ibe.med.uni-muenchen.de; 4 Department of Pneumology and Respiratory Medicine, Hospital Schillerhoehe, Academic Teaching Hospital of the University of Tuebingen, 70376 Gerlingen, Germany; claus.neurohr@rbk.de

**Keywords:** interstitial lung disease, idiopathic pulmonary fibrosis, physical activity, cough

## Abstract

Physical activity limitations and cough are common in patients with interstitial lung disease (ILD), potentially leading to reduced health-related quality of life. We aimed to compare physical activity and cough between patients with subjective, progressive idiopathic pulmonary fibrosis (IPF) and fibrotic non-IPF ILD. In this prospective observational study, wrist accelerometers were worn for seven consecutive days to track steps per day (SPD). Cough was evaluated using a visual analog scale (VAS_cough_) at baseline and weekly for six months. We included 35 patients (IPF: *n* = 13; non-IPF: *n* = 22; mean ± SD age 61.8 ± 10.8 years; FVC 65.3 ± 21.7% predicted). Baseline mean ± SD SPD was 5008 ± 4234, with no differences between IPF and non-IPF ILD. At baseline, cough was reported by 94.3% patients (mean ± SD VAS_cough_ 3.3 ± 2.6). Compared to non-IPF ILD, patients with IPF had significantly higher burden of cough (*p* = 0.020), and experienced a greater increase in cough over six months (*p* = 0.009). Patients who died or underwent lung transplantation (*n* = 5), had significantly lower SPD (*p* = 0.007) and higher VAS_cough_ (*p* = 0.047). Long-term follow up identified VAS_cough_ (HR: 1.387; 95%-CI 1.081–1.781; *p* = 0.010) and SPD (per 1000 SPD: HR 0.606; 95%-CI: 0.412–0.892; *p* = 0.011) as significant predictors for transplant-free survival. In conclusion, although activity didn’t differ between IPF and non-IPF ILD, cough burden was significantly greater in IPF. SPD and VAS_cough_ differed significantly in patients who subsequently experienced disease progression and were associated with long-term transplant-free survival, calling for better acknowledgement of both parameters in disease management.

## 1. Introduction

Fibrotic interstitial lung diseases are severe conditions in which a range of symptoms restrict patients’ everyday lives. Beyond dyspnea and impaired respiratory capacity [1], other factors can have an impact on these patients’ outcomes and health-related quality of life (HRQoL). This includes, for example, chronic cough and restricted daily activities, although, so far, most of the data relates to patients with idiopathic pulmonary fibrosis (IPF), with limited information on other non-IPF fibrotic ILDs. Primarily a consequence of decreased lung function and breathlessness, physical activity is reduced in patients with fibrotic ILDs, including IPF [2,3,4,5]. Many patients with chronic lung disease such as fibrotic ILDs avoid exercise due to a fear of worsening lung function or dyspnea during exercise [6]. This is of particular concern in ILD since staying physically active is very important, with, for example, a reduction in physical activity associated with increased risk of mortality in IPF [2]. Nevertheless, it is likely that various parameters could influence physical activity and inactivity in these patients, as for example pain has been shown to be an independent predictor of decreased activity in fibrotic ILD [7]. Another such factor might be chronic cough [1,8]. Many patients with ILD (especially fibrotic ILD) suffer from chronic cough, in turn significantly impacting HRQoL [9], and potentially also physical activity. Cough is present in up to 84% of patients with IPF [10], but the pathophysiology is complex and still poorly understood with limited therapy options [11], and limited data available on cough in non-IPF fibrotic ILD.

We are not aware of studies simultaneously investigating both physical activity and cough in fibrotic ILD, comparing IPF and non-IPF ILD. Therefore, we aimed to characterize the differences in cough and physical activity between patients with IPF and those with non-IPF fibrotic ILD, and to evaluate their relationship with HRQoL.

## 2. Materials and Methods

### 2.1. Study Design and Cohort

In this prospective observational study, physical activity and cough were assessed in patients with subjective, progressive fibrotic ILD using accelerometers and self-report questionnaires. In our study, the definition of “progressive fibrotic ILD” was defined as any subjective increase in dyspnea and/or physical limitations within the last six months. An acute infection, heart failure, atrial fibrillation or anaemia causing the subjective deterioration was excluded prior to inclusion in the study. Progression did not include worsening of pulmonary function and/or increase of fibrosis on high-resolution computed tomography (HRCT). Patients were recruited between July 2017 and August 2018 in the in- and out-patient unit of the Department of Internal Medicine V of the University of Munich, and were followed for 6 months. The study was conducted in accordance with the amended Declaration of Helsinki and approved by the local ethics committee of the University of Munich, Germany and (UE No. 812-16). All patients provided written informed consent.

We included individuals with a consensus diagnosis of fibrotic ILD, including IPF, chronic hypersensitivity pneumonitis, connective tissue disease-related ILD (CTD-ILD), non-specific interstitial pneumonia (NSIP) and unclassifiable pulmonary fibrosis. All diagnoses were made based on multidisciplinary discussion of the diagnosis in accordance with the current international criteria [12,13,14]. Medical history, current medication, and relevant comorbidities at the time of study inclusion were collected. Lung function testing (spirometry, plethysmography and gas transfer) and clinical assessments (including 6-min walking distance [6MWD]) were performed at baseline. Although not a specific exclusion criterion, none of the study patients had completed pulmonary rehabilitation within 6 months prior to recruitment or took part in such a program during the study period. 

Patients were followed prospectively until disease progression, or on completion of a follow-up-duration of 6 months. Disease progression was defined as lung transplantation, or death due to respiratory failure. Long-term follow-up was evaluated until 30 April 2023.

### 2.2. Physical Activity

Each patient was given 30 min dedicated instruction on how to use the wristband accelerometer (Polar A360 Fitness Tracker, Polar Electro GmbH, Büttelborn, Germany), which was initialized with personal settings (age, sex, weight and height) and then worn on the nondominant wrist for seven consecutive days at baseline. Patients were instructed to follow their daily, normal physical activity level, and only remove the monitors for sleep during nighttime. The activity monitors were only worn for 7 consecutive days at baseline. Data were recorded on the device, which was sent back to the hospital after 7 days, where data were read out and analyzed using the Polar Flowsync software version 2017 and 2018 (Polar Electro GmbH, Büttelborn, Germany). Accelerometer data were considered usable for each day if the patient had worn the device for more than 10 h. Recorded activity was categorized as: Lying, sitting, standing, walking, or running. Based on these data, activity time was defined as the sum of standing, walking and running time. In addition, wearing time of the accelerometer, steps per day (SPD) and distance covered (in meters) were recorded and analyzed. Only patients with at least 4 days on which the device was worn for more than 10 h per day during the 7 consecutive days were included in the final analysis.

### 2.3. Cough

Patients were instructed to complete a visual analog scale (VAS_cough_) for cough with higher values indicating greater burden of cough [15]. Therefore, patients were asked to document their subjective burden of cough by making a handwritten mark on a 10-cm line, which symbolized a continuum between “no cough” (0 mm) and “worst cough ever” (100 mm). The VAS_cough_ has been shown as to be a valid tool assessing cough severity in patients with chronic cough [16]. Evaluation of VAS_cough_ was done at baseline and after 3 and 6 months in the hospital, with patients also requested to fill out a VAS_cough_ weekly over 6 months.

### 2.4. Questionnaires Assessing Health-Related Quality of Life

The St. George’s Respiratory Questionnaire (SGRQ), which was designed as a clinical research tool to study overall health, daily life, and perceived well-being in patients with chronic airways disease [17], has been subsequently validated and applied as a measure of HRQoL patients with IPF [18,19]. Scores range from 0 to 100, with higher scores indicating greater limitations. The ILD-specific King’s Brief Interstitial Lung Disease questionnaire (KBILD) consists of questions covering the three domains breathlessness and activities, psychological aspects, and chest symptoms [20], with scores correlating with respiratory parameters in ILD [21]. KBILD is a 15-item validated questionnaire, in which higher scores indicate fewer limitations. Patients completed the SGRQ and the K-BILD questionnaire at baseline, and after 3 and 6 months [20,22,23].

### 2.5. Statistical Analysis

Continuous variables are presented as the mean ± standard deviation (SD), with categorical variables summarized by frequency and percentage. The Mann-Whitney test was used to compare continuous variables, the Chi square and Fisher’s exact tests were used to compare categorical variables, and Pearson’s correlation test identified correlations between parameters. To calculate VAS_cough_ change over the follow-up period, a linear regression model was used with all available parameters without imputation. Individual VAS_cough_ variation was calculated using the coefficient of variation (CoV) of all VAS_cough_ values within the first three months. To evaluate long-term effects in the study cohort, retrospectively, follow-up data was collected until 30 April 2023. The Kaplan–Meier method was used to evaluate transplant-free survival time in the study population over 6 months and the long-term follow up, with a log-rank test used to analyze differences between groups (IPF versus non-IPF ILD). Further, a cox proportional hazard regression analysis was performed and included retrospective follow-up until 30 April 2023. The cox proportional hazard regression analysis was applied to identify associations of VAS_cough_ and SPD with transplant-free survival time including the covariates baseline FVC and IPF. In the Cox proportional hazard regression analysis SPD were analyzed per 1000 SPD. *p* values < 0.05 were considered statistically significant. Data were statistically analyzed by SPSS version 25.0 and 29.0 (IBM SPSS, Armonk, NY, USA) or GraphPad Prism version 8.0 for Mac (GraphPad Software, San Diego, CA, USA), with *p* < 0.05 considered statistically significant.

## 3. Results

### 3.1. Study Cohort

**Figure 1:**
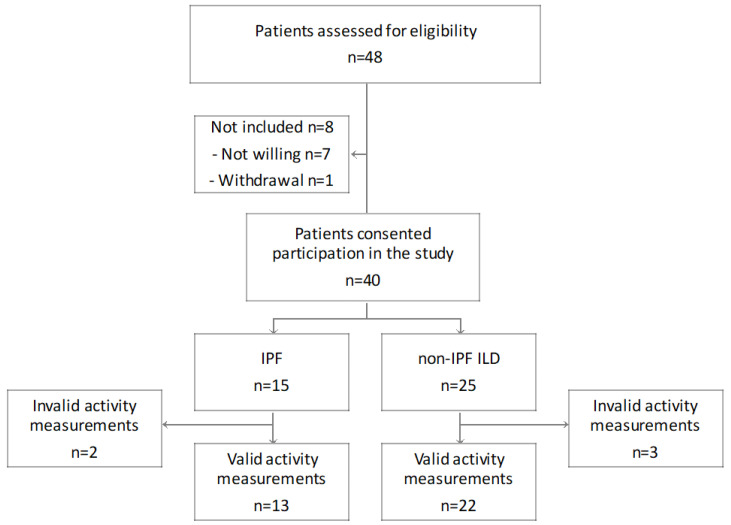
Study cohort. Validity of activity measurements was defined as at least four days, where a valid day was a wearing time of the device >10 h per day. Abbreviations: Idiopathic pulmonary fibrosis (IPF), interstitial lung disease (ILD).

From July 2017 until August 2018, 40 patients consented to participate in the study. Since five patients had fewer than 4 days of activity measurements, only 35 patients (IPF *n* = 13; non-IPF *n* = 22) were included in the analyses as depicted in Figure 1. Non-IPF ILD conditions included CTD-ILD (*n* = 7), chronic hypersensitivity pneumonitis (*n* = 7), unclassifiable ILD (*n* = 5) and NSIP (*n* = 3). 

The majority of patients had severe lung function impairment, as indicated by reduced FVC and DLCO % predicted. Only 20 patients (57.1%) were able to perform a DLCO measurement. Patients’ characteristics are summarized in Table 1; the demographic characteristics and disease severity were similar in the IPF and non-IPF ILD subgroups. At the beginning of the study 84.6% (*n* = 11) patients with IPF were receiving antifibrotic therapy. Among patients with non-IPF ILD 72.7% (*n* = 16) were receiving immunosuppressive therapy and 22.7% (*n* = 5) were receiving antifibrotic therapy. There were no differences between the IPF and non-IPF ILD groups in terms of comorbidities (Table 2). The mean ± SD follow-up time in the cohort was 163 ± 39 days.

At baseline, mean KBILD was 52.4 ± 11.3 and mean SGRQ was 49.2 ± 18.7, with the measures correlating moderately with each other (r = –0.400; *p* = 0.023). Comparing patients with IPF and those with non-IPF ILD, patients with IPF had significantly more limitations in terms of KBILD values compared to those with non-IPF ILD (48.2 ± 2.6 vs. 53.1 ± 12.1; *p* = 0.022; Table 1). The SGRQ did not show significant differences between IPF and non-IPF ILD (51.1 ± 9.8 vs. 48.9 ± 20.0; *p* = 0.193). Over time, neither KBILD nor SGRQ changed significantly in non-IPF ILD or IPF.

### 3.2. Physical Activity

The mean ± SD wearing time of the wristband accelerometer was 14.7 ± 2.4 h per day. Our cohort had a mean of 5008 ± 4234 SPD at baseline (min 678, max 18504; median 3757), with distance covered 2874 ± 2564 m, active time (standing, walking and running) 4.8 ± 2.4 h/day, and calorie expenditure of 2167 ± 521 kcal calculated by the device. SPD correlated strongly with the distance covered (r = 0.97; *p* < 0.001), and moderately with the active time (r = 0.42; *p* = 0.012) and calorie expenditure (r = 0.37; *p* = 0.031). Additionally, active time, calorie expenditure, and the distance covered correlated with each other (active time and calorie expenditure: r = 0.742; *p* < 0.001; active time and distance covered: r = 0.632; *p* < 0.001; calorie expenditure and distance covered: r = 0.367, *p* = 0.030).

**Figure 2:**
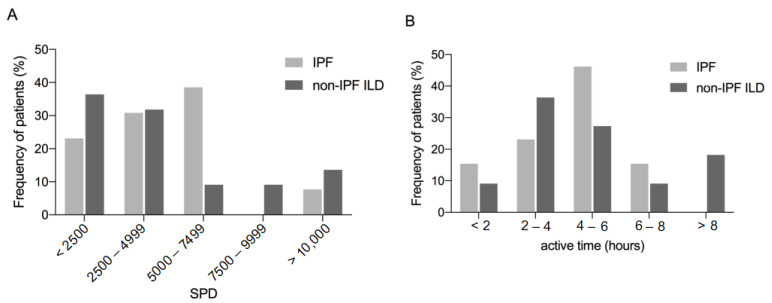
Physical activity in patients with ILD. Physical activity measured by wrist accelerometer in patients with ILD displayed as frequency of daily steps (step per day; SPD) (**A**) and active time (walking and running time; (**B**)). Abbreviations: Idiopathic pulmonary fibrosis (IPF), interstitial lung disease (ILD), steps per day (SPD).

There were no differences between patients with IPF and non-IPF ILD in wearing time (*p* = 0.987), SPD (*p* = 0.489; Figure 2A), distance covered (*p* = 0.489), calorie expenditure (*p* = 0.827) or active time (*p* = 0.933; Figure 2B). Over 50% of the cohort averaged fewer than 5000 SPD (Figure 2A). SPD correlated with the clinical parameters FVC % predicted, 6MWD and BMI in all patients and also separately in the IPF and non-IPF ILD cohorts as shown in Table 3. KBILD, but not the SGRQ correlated significantly with SPD in the whole study group (Table 3). Neither the active time nor consumed calories correlated with clinical parameters FVC % predicted, DLCO, 6MWD or BMI in IPF or non-IPF ILD.

### 3.3. Cough

At baseline, cough was present in 94.3% of the patients (*n* = 33; IPF: 100%; non-IPF: 91.3%) with mean ± SD cough burden as measured with the VAS_cough_ of 3.3 ± 2.6 (range 0 to 8.8). There was no difference in VAS_cough_ between patients with and without gastroesophageal reflux disease (*p* = 0.12). For KBILD, but not SGRQ, there was a significant inverse correlation with VAS_cough_ (Table 4).

**Figure 3:**
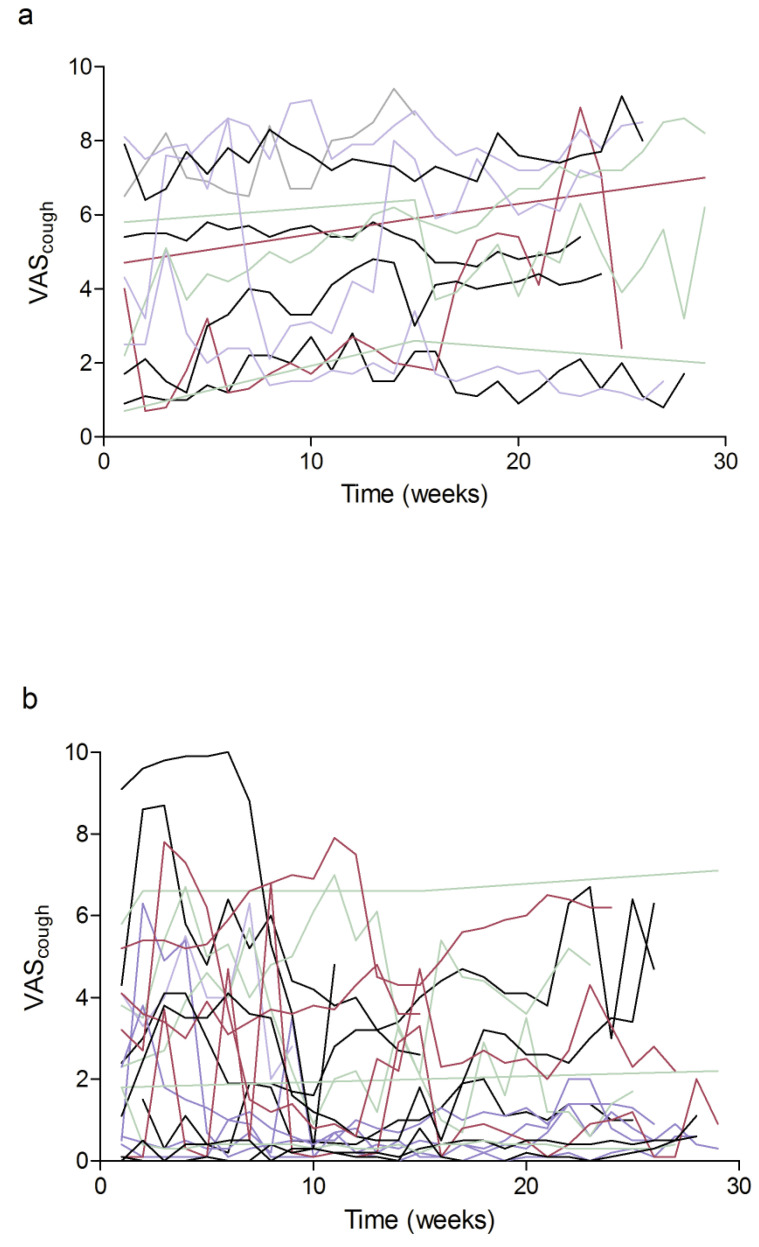
Individual weekly VAS_cough_ in the study cohort. Lines show individual course of VAS_cough_ in patients in the (**a**) IPF and (**b**) non-IPF ILD subgroups over six months. Abbreviations: visual analogue scale (VAS), idiopathic pulmonary fibrosis (IPF), interstitial lung disease (ILD).

Patients with IPF had a mean ± SD VAS_cough_ of 4.6 ± 2.7 at baseline. During the study, a mean of 20.6 ± 8.9 VAS_cough_ measurements were recorded by patients with IPF, with cough increasing over the six months (+1.2 ± 1.9 VAS_cough_; Figure 3a). Within the first 3 months, the variability of VAS_cough_, represented by CoV, was 27.7 ± 20.6%. At individual patient level, there was a significant inverse correlation between VAS_cough_ at baseline and CoV (r = –0.710; *p* = 0.007), although change in VAS_cough_ over 6 months was not associated with VAS_cough_ at baseline (r = –0.191; *p* = 0.574).

In non-IPF ILD, the mean ± SD VAS_cough_ was 2.5 ± 2.4 at baseline. A mean ± SD of 20.8 ± 8.8 VAS_cough_ measurements were obtained over 6 months, with an overall decrease in mean VAS_cough_ (–0.7 ± 1.0 VAS_cough_; Figure 3b), with CoV over the first three months of 65.6 ± 44.5%. As in IPF, there was a significant inverse correlation between VAS_cough_ at baseline and CoV at individual patient level (r = –0.581; *p* = 0.006), and the change of VAS_cough_ over 6 months was independent from VAS_cough_ at baseline (r = –0.030; *p* = 0.910).

When comparing the findings on VAS_cough_ in IPF and non-IPF ILD, we identified marked differences between the two groups. Baseline VAS_cough_ was significantly higher in IPF than in non-IPF ILD (*p* = 0.020). While no association between SPD or active time and cough could be found in any group, in non-IPF ILD but not in IPF VAS_cough_ showed a significant association to FVC, DLCO and the 6MWD (Table 4). In addition, over the 6 months the slopes of VAS_cough_ increased in IPF but decreased in non-IPF ILD, with a significant difference in the trajectories (*p* = 0.009). Further, the variation of VAS_cough_ was significantly lower in patients with IPF than in those with non-IPF ILD (*p* = 0.012).

### 3.4. Transplant-Free Survival

**Figure 4:**
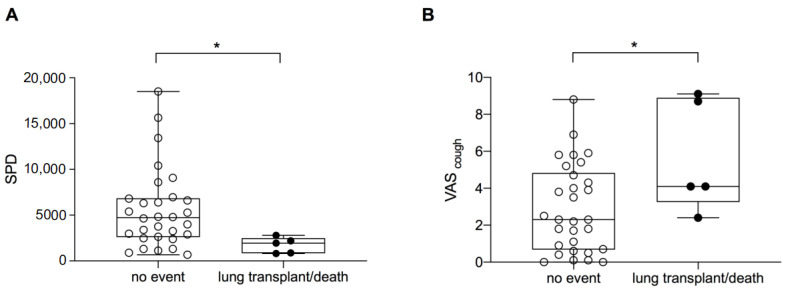
SPD and VAS_cough_ in progressive ILD. SPD (**A**) were significantly lower in patients with disease progression (*p* = 0.007) and VAS_cough_ (**B**) was significantly higher in patients with death due to respiratory failure and lung transplantation (*p* = 0.047). * indicates a *p*-value < 0.05; ● indicates patients who died or underwent lung transplantation within six months; ◦ indicates patients without such an event; Abbreviations: steps per day (SPD), visual analogue scale (VAS), interstitial lung disease (ILD).

Over six months, two patients died due to respiratory failure (IPF *n* = 1; non-IPF ILD *n* = 1) and three patients underwent lung transplantation (all in the non-IPF group). While there were no significant differences in age (*p* = 0.91), FVC (*p* = 0.24), DLCO (*p* = 0.88) or active time (*p* = 0.09) between patients who died or underwent lung transplantation vs. the remaining 30 patients, mean SPD was significantly lower (1719 ± 861 vs. 5557 ± 4329; *p* = 0.007; Figure 4A) and VAS_cough_ was significantly higher in these five patients (5.7 ± 3.0 vs. 2.9 ± 2.4; *p* = 0.047; Figure 4B). Of note, the five patients who died or underwent lung transplantation had significantly lower KBILD values at baseline (41.4 ± 4.9 vs. 54.2 ± 11.1; *p* = 0.002) and significantly lower SGRQ (37.4 ± 6.9 vs. 51.2 ± 19.4; *p* = 0.044). Transplant-free survival did not differ between patients with IPF and non-IPF ILD within the first 6 months (*p* = 0.433; mean transplant-free survival IPF: 189 ± 13 days; non-IPF ILD: 207 ± 11 days).

During the long-term follow up, 17.1% of the patients died due to respiratory failure (IPF *n* = 3; non-IPF ILD *n* = 3), 28.6% of the patients underwent lung transplantation (IPF *n* = 4; non-IPF ILD *n* = 6) and 17.1% of the patients were lost to follow-up (IPF *n* = 3; non-IPF ILD *n* = 3). The mean ± SD transplant-free survival time was 1342 ± 145 days (IPF: 1218 ± 223 days; non-IPF ILD: 1395 ± 188 days; *p* = 0.549). In the regression analysis VAS_cough_ and SPD (as calculated per every 1000 SPD) were independent predictors for transplant-free survival (VAS_cough_: HR 1.387; 95% CI: 1.081–1.781; *p* = 0.010; SPD (per 1000 SPD) HR: 0.606; 95%-CI; *p* = 0.011) including the covariates baseline FVC and IPF (Table 5).

## 4. Discussion

Reduced physical activity and cough can lead to significant limitations in life in patients with fibrotic ILD. In our study, we have demonstrated that the level of physical activity did not differ between patients with IPF and those with fibrotic non-IPF ILD, and that in both groups reduced physical activity was associated with more limited respiratory capacity. In contrast, we identified marked differences between IPF and non-IPF ILD in terms of cough: Patients with IPF not only had a higher burden of cough at the beginning of the study, but also experienced a greater increase in cough over time than those with non-IPF ILD. In addition, at baseline both VAS_cough_ and SPD differed significantly between the patients who subsequently experienced death or lung transplantation during the study period and those without such an event.

A main focus of our study was daily physical activity, which has been shown to be markedly reduced in advanced ILD [5]. We identified significant correlations of SPD with clinical parameters such as FVC, 6MWD and BMI, not only in the overall cohort but also when analyzing IPF and non-IPF ILD separately. Lung function parameters and 6MWD have previously been shown to be associated with SPD, both in patients with IPF and in those with idiopathic interstitial pneumonia supporting our findings [3,24]. Furthermore, SPD is a predictor of mortality in IPF [2]. Consistent with this previous analysis, we also found SPD to be significantly and markedly reduced in patients who subsequently died or underwent lung transplantation during the six months study period. Moreover, SPDs were a significant predictor for long-term transplant-free survival in our cohort. Since rehabilitation programs have been shown to have a positive impact on physical activity [5], patients with fibrotic ILD should take part in such controlled programs, with benefits potentially including an increase in HRQoL. As we showed that there were no differences in physical activity parameters between the IPF and non-IPF groups, both groups would profit from such programs. In terms of routine patient care, daily activity monitoring with a wrist-based accelerometer could easily be implemented outside of clinical trials to assess physical activity in patients with ILD.

While it has been shown that cough is a significant, distressing symptom in IPF [9], clinical management still needs improvement. Although most studies of cough in ILD focus on patients with IPF, cough and its impact on HRQoL has also been described in patients with chronic hypersensitivity pneumonitis [8], sarcoidosis [25] and systemic sclerosis [8,26,27]. We observed significant differences between patients with IPF and those in the non-IPF ILD subgroup: the burden of cough was higher in IPF; and patients with IPF experienced an increase in cough burden over the study period. To our knowledge, our study is the first using VAS_cough_ to evaluate cough longitudinally in ILD. This longitudinal observation allows us to gain new insights into cough in ILD, such as short-term varying burden of cough. Interestingly, while patients with non-IPF ILD had greater variability in cough, patients with IPF had more consistent burden of cough. Furthermore, VAS_cough_ at baseline was significantly higher in patients who died or underwent lung transplantation during the study. In our cox regression analysis, higher VAS_cough_ was a significant predictor for transplant-free survival. Our findings support those of an earlier study in patients with IPF that identified cough as being more prevalent in advanced disease, and was borderline associated with decreased transplant-free survival time [10]. In our study, in the non-IPF ILD, but not in the IPF subgroup VAS_cough_ correlated with more reduced lung function parameters (FVC and DLCO). This could be due to a greater overall burden of cough in IPF, which is already present in less advanced disease. Our data support the need to develop strategies to better alleviate cough (including new therapeutic agents), and to incorporate cough management in ILD care strategies [11]. Given the greater subjective burden of cough in patients with fibrotic ILD, these patients should be regularly questioned about coughing. The VAS_cough_ is a standardized, easy-to-collect instrument that could quickly be integrated into clinical routine.

To evaluate the impact of cough and activity on HRQoL, we used the two questionnaires KBILD and SGRQ. Overall, mean baseline KBILD was similar to a previously published study in patients with ILD at an outpatient clinic [28]. KBILD correlated with SPD, active time and VAS_cough_. The correlation with cough seems to be robust as it was not only seen at baseline but also at the 3 months visit. Although KBILD and SGRQ correlated moderately at baseline, the relationships between KBILD and cough and activity were not observed for SGRQ. This contrasts with another study, which identified an association between SPD and SGRQ [24]. The reasons why there are conflicting results with this previous study concerning the correlation between the SGRQ and SPD remain unclear. Whether this could be associated with our transplant-center enriched patient population (younger age and likely less comorbidities) needs to be further established. Also, the differences in HRQoL between IPF and non-IPF ILD that were detected by KBILD were not seen using SGRQ. This could be an indication that questionnaires specifically designed for ILD, such as KBILD or a recently published IPF-specific version of the SGRQ [29], are better suited to capture HRQoL in ILD than the original SGRQ. 

We acknowledge the inherent limitations of our study. First, this was a single-center study with a limited number of patients. Further, our patients were already in an advanced stage of disease, which could be one reason for the very high prevalence of cough. Moreover, our accelerometer was not an existing research instrument: To measure physical activity, we used an ordinary fitness tracker. Our device was one of the five most often used fitness tracker (wrist-worn) brands in research projects [30]. We are not aware of previous studies using such a device to monitor physical activity in ILD. Nevertheless, the results of our accelerometer were comparable with previously published data on SPD in IPF and other fibrotic ILD [7,24]. Finally, in our study the activity tracker was only worn for 7 days at baseline. This could potentially result in a trial effect, and an artificial increase of activity levels (from patients being able to see their data). Nevertheless, this study design has been used before to evaluate physical activity [3,24]. Finally, in this study, patients with fibrotic ILD and subjective clinical progression were included. At the time when the study was conducted there was no official definition for a progressive phenotype of pulmonary fibrosis. Further, antifibrotic therapy in non-IPF ILD was not common outside of clinical trails which explains why only 22.7% of the non-IPF ILD patients were on antifibrotic therapy. In recent years new international und national guidelines have been published proposing criteria of progression in fibrotic ILD and recommend an antifibrotic therapy in these patients [31,32]. In 12 patients (IPF *n* = 6; non-IPF ILD *n* = 6) the subjective progression of ILD was accompained by a FVC decline of >5% absolute over the last year, retrospectively indicating a pogressive phenotype according to the current guideline [31]. Interestingly, in these patients, neither SPD nor VAS_cough_ at baseline showed significant differences compared to the other patients. The limited number of patients, the lung transplants and the advanced stage of the diseases could explain why the transplant-free survival of patients with IPF was not significantly different from that of patients with non-IPF ILD.

## 5. Conclusions

In conclusion, while physical activity does not differ between IPF and non-IPF ILD, there were marked differences in cough between the two groups. Cough was present in over 90% of all patients with advanced ILD, with the VAS_cough_ correlating with DLCO, and patients who subsequently died or underwent lung transplantation having higher cough burden. In particular, patients with IPF reported more cough, which increased over time. While we did not observe an association between cough and physical activity, SPD significantly correlated with lung function and exercise capacity and was significantly reduced in patients who subsequently died or underwent lung transplantation. Moreover, VAS_cough_ and SPD were independent predictors for transplant-free survival in the study cohort. Both cough and physical activity should be key components in disease management.

## Figures and Tables

**Table 1:** Baseline characteristics of study cohort.

	All (*n* = 35)	IPF (*n* = 13)	Non-IPF ILD (*n* = 22)	*p*-Value
Age (years)	61.8 ± 10.8	65.3 ± 7.2	59.8 ± 12.1	0.145
Sex (male), *n* (%)	20 (57.1)	10 (76.9)	10 (45.5)	0.069
BMI (kg/m^2^)	27.7 ± 4.2	27.0 ± 4.7	28.1 ± 4.0	0.471
Smoking status				
Non-smokers	16 (45.7)	3 (23.1)	13 (59.1)	0.08
Ex-smoker	19 (54.3)	10 (76.9)	9 (40.9)	0.08
Current smokers	0	0	0	1.0
Smoking history (py)	21.4 ± 13.4	23.5 ± 11.3	19.0 ± 15.7	0.479
Lung function				
FVC, % predicted	65.3 ± 21.7	68.5 ± 18.7	63.4 ± 23.5	0.507
FVC (L)	2.4 ± 0.8	2.7 ± 0.8	2.2 ± 0.8	0.135
TLC, % predicted	70.5 ± 20.4	70.5 ± 19.1	70.5 ± 21.6	0.991
TLC (L)	4.2 ± 1.1	4.6 ± 1.2	4.0 ± 1.0	0.210
DLCO, % predicted ^a^	42.8 ± 16.4	38.9 ± 11.5	45.4 ± 19.1	0.427
6MWD (m) ^b^	381.3 ± 125.7	431.2 ± 89.5	353.9 ± 136.0	0.102
KBILD	52.4 ± 11.3	48.2 ± 2.6	53.1 ± 12.1	0.022
SGRQ	49.2 ± 18.7	51.1 ± 9.8	48.9 ± 20.0	0.193

Data are presented as mean ± SD and number and percentage, respectively. *p* values are for the comparison of the IPF and non-IPF groups. Abbreviations: Idiopathic pulmonary fibrosis (IPF), interstitial lung disease (ILD), body mass index (BMI), pack years (py), forced vital capacity (FVC), total lung capacity (TLC), diffusion capacity for carbon monoxide (DLCO), 6-min walk distance (6MWD), King’s Brief Interstitial Lung Disease questionnaire (KBILD), St. George’s Respiratory Questionnaire (SGRQ). ^a^ Available in 20 patients (8/12 in the IPF/non-IPF groups, respectively). ^b^ Available in 31 patients (11/20).

**Table 2:** Comorbidities in the study cohort.

Comorbidity	All (*n* = 35)	IPF (*n* = 13)	Non-IPF ILD (*n* = 22)	*p*-Value
Arterial hypertension, *n* (%)	16 (45.7)	6 (46.2)	10 (45.5)	0.968
Diabetes mellitus, *n* (%)	4 (11.4)	2 (15.4)	2 (9.1)	0.572
Hyperlipoproteinemia, *n* (%)	3 (8.6)	2 (15.4)	1 (4.5)	0.268
Coronary artery disease, *n* (%)	8 (22.9)	1 (7.7)	7 (31.8)	0.101
History of revascularization, n (%)	5 (14.3)	1 (7.7)	4 (18.2)	0.392
Osteoporosis, *n* (%)	3 (8.6)	1 (7.7)	2 (9.1)	0.886
Obstructive sleep apnea syndrome, *n* (%)	4 (8.7)	2 (15.4)	2 (9.1)	0.572
Gastroesophageal reflux disease, *n* (%)	8 (22.9)	3 (23.1)	5 (22.7)	0.981
Atrial fibrillation, *n* (%)	1 (4.3)	0 (0)	1 (4.5)	0.435
Pulmonary hypertension, *n* (%) ^a^	9 (25.7)	3 (23.1)	6 (27.3)	0.926

Data are presented as number and percentage, respectively. *p* values are for the comparison of the IPF and non-IPF groups. ^a^ Available in 28 patients (9/19). Abbreviations: Idiopathic pulmonary fibrosis (IPF), interstitial lung disease (ILD).

**Table 3:** Bivariate correlations between steps per day (SPD) and clinical parameters in ILD.

	All		IPF		Non-IPF-ILD	
	r	*p*-Value	r	*p*-Value	r	*p*-Value
FVC, % predicted	0.495	0.003	0.706	0.007	0.464	0.03
DLCO, % predicted	0.323	0.165	0.440	0.276	0.282	0.375
6MWD, m	0.537	0.002	0.695	0.018	0.518	0.019
BMI (kg/m^2^)	−0.050	0.776	−0.185	0.545	−0.019	0.934
KBILD	0.353	0.038	0.067	0.827	0.405	0.062
SGRQ	−0.56	0.755	0.414	0.160	−0.131	0.571

Bivariate correlations with Pearson’s correlation coefficient r between physical activity (steps per day; SPD) and clinical variables. Abbreviations: Idiopathic pulmonary fibrosis (IPF), interstitial lung disease (ILD), steps per day (SPD), forced vital capacity (FVC), diffusion capacity for carbon monoxide (DLCO), 6-min walk distance (6MWD), body mass index (BMI). King’s Brief Interstitial Lung Disease questionnaire (KBILD), St. George’s Respiratory Questionnaire (SGRQ).

**Table 4:** Bivariate correlations between VAScough and clinical parameters in ILD.

	All		IPF		Non-IPF-ILD	
	r	*p*-Value	r	*p*-Value	r	*p*-Value
FVC, % predicted	−0.222	0.119	0.107	0.725	−0.478	0.025
DLCO, % predicted	−0.466	0.038	−0.148	0.727	−0.640	0.025
6MWD, m	−0.367	0.042	0.144	0.738	−0.773	<0.01
SPD	−0.227	0.189	0.086	0.779	−0.350	0.110
Active time	−0.207	0.232	−0.090	0.771	−0.221	0.324
KBILD	−0.539	0.001	−0.616	0.025	−0.437	0.042
SGRQ	0.195	0.269	−0.063	0.838	0.204	0.374

Bivariate correlations with Pearson’s correlation coefficient r between VAScough and clinical variables. Abbreviations: visual analogue scale (VAS), idiopathic pulmonary fibrosis (IPF), interstitial lung disease (ILD), forced vital capacity (FVC), diffusion capacity for carbon monoxide (DLCO), 6-min walk distance (6MWD), steps per day (SPD), King’s Brief Interstitial Lung Disease questionnaire (KBILD), St. George’s Respiratory Questionnaire (SGRQ).

**Table 5:** Cox proportional hazard regression analysis assessing the effect on transplant-free survival.

	Hazard Ratio	95%–CI	*p*-Value
Baseline FVC, % predicted	0.973	0.941–1.007	0.119
IPF	1.708	0.569–5.125	0.340
VAS_cough_	1.387	1.081–1.781	0.010
SPD (per 1000 SPD)	0.606	0.412–0.892	0.011

The analyzes for SPD were calculated per 1000 SPD. Abbreviations: FVC, forced vital capacity, idiopathic pulmonary fibrosis (IPF), visual analogue scale (VAS), steps per day (SPD), confidence interval (CI).

## Data Availability

The data is unavailable due to privacy restrictions.
